# Neurodegeneration Induced by Anti-IgLON5 Antibodies Studied in Induced Pluripotent Stem Cell-Derived Human Neurons

**DOI:** 10.3390/cells10040837

**Published:** 2021-04-08

**Authors:** Matias Ryding, Mattias Gamre, Mette S. Nissen, Anna C. Nilsson, Justyna Okarmus, Anne A. E. Poulsen, Morten Meyer, Morten Blaabjerg

**Affiliations:** 1 Department of Neurobiology Research, Institute of Molecular Medicine, University of Southern Denmark, 5000 Odense, Denmark; mknudsen@health.sdu.dk (M.R.); anders.mattias.wallumrod.gamre@rsyd.dk (M.G.); jokarmus@health.sdu.dk (J.O.); mmeyer@health.sdu.dk (M.M.); 2 Department of Neurology, Odense University Hospital, 5000 Odense, Denmark; mette.scheller.nissen2@rsyd.dk (M.S.N.); anpou17@student.sdu.dk (A.A.E.P.); 3 Department of Clinical Research, Odense University Hospital, 5000 Odense, Denmark; 4 Department of Clinical Immunology, Odense University Hospital, 5000 Odense, Denmark; anna.christine.nilsson@rsyd.dk; 5 BRIDGE—Brain Research Inter-Disciplinary Guided Excellence, Department of Clinical Research, University of Southern Denmark, 5000 Odense, Denmark

**Keywords:** autoimmune encephalitis, IgLON5, phosphorylated tau, neurodegeneration

## Abstract

Anti-IgLON5 disease is a progressive neurological disorder associated with autoantibodies against a neuronal cell adhesion molecule, IgLON5. In human postmortem brain tissue, the neurodegeneration and accumulation of hyperphosphorylated tau (p-tau) are found. Whether IgLON5 antibodies induce neurodegeneration or neurodegeneration provokes an immune response causing inflammation and antibody formation remains to be elucidated. We investigated the effects of anti-IgLON5 antibodies on human neurons. Human neural stem cells were differentiated for 14–48 days and exposed from Days 9 to 14 (short-term) or Days 13 to 48 (long-term) to either (i) IgG from a patient with confirmed anti-IgLON5 antibodies or (ii) IgG from healthy controls. The electrical activity of neurons was quantified using multielectrode array assays. Cultures were immunostained for β-tubulin III and p-tau and counterstained with 4′,6-Diamidine-2′-phenylindole dihydrochloride (DAPI). To study the impact on synapses, cultures were also immunostained for the synaptic proteins postsynaptic density protein 95 (PSD95) and synaptophysin. A lactate dehydrogenase release assay and nuclei morphology analysis were used to assess cell viability. Cultures exposed to anti-IgLON5 antibodies showed reduced neuronal spike rate and synaptic protein content, and the proportion of neurons with degenerative appearance including p-tau (T205)-positive neurons was higher when compared to cultures exposed to control IgG. In addition, cell death was increased in cultures exposed to anti-IgLON5 IgG for 21 days. In conclusion, pathological anti-IgLON5 antibodies induce neurodegenerative changes and cell death in human neurons. This supports the hypothesis that autoantibodies may induce the neurodegenerative changes found in patients with anti-IgLON5-mediated disease. Furthermore, this study highlights the potential use of stem cell-based in vitro models for investigations of antibody-mediated diseases. As anti-IgLON5 disease is heterogeneous, more studies with different IgLON5 antibody samples tested on human neurons are needed.

## 1. Introduction

Anti-IgLON5 disease is a novel neurological disorder, thought to be autoantibody mediated, but with a distinct neurodegenerative pattern on postmortem tissue [1,2]. It was initially recognized as a sleep disorder with nonrapid eye movement (NREM) and REM parasomnia, breathing dysfunction, gait instability, and bulbar symptoms, but it is now known to be more clinically heterogeneous in its presenting symptomatology and response to immunotherapy [1,3,4,5]. The disease affects men and women equally and is insidious in onset, with slow progression over several years and an often fatal outcome [3]. Postmortem analyses have revealed neuronal deposits of hyperphosphorylated tau (p-tau) protein in the hypothalamus and the brainstem tegmentum [2]. This deposition, consisting of three and four repeat isoforms, has a different distribution compared to other neurodegenerative conditions, suggesting anti-IgLON5 disease to be a novel tauopathy [1,6,7]. Despite the neurodegenerative features, a strong association with human leukocyte antigen haplotypes (HLA-DRB1*10:01 and HLA-DQB1*05:01) and a response to immunotherapy in some cases indicates an immunological disease component [4,8]. The clinical manifestations of the disease were initially divided into four main phenotypes: (1) predominant sleep disorder, (2) bulbar syndrome, (3) Progressive Supranuclear Palsy-like syndrome, and (4) cognitive impairment that may associate chorea [3]. Recently, isolated gait ataxia and nervous system hyperexcitability have been included in the spectrum [9]. The sleep disorder is characterized by altered NREM sleep initiation, vocalizations, and finalistic movements (NREM parasomnia) [1,10]. REM behavior disorder and sleep-breathing difficulties such as obstructive sleep apnea and stridor can present early or late but are present in a majority of patients [10]. The diagnosis relies on the clinical presentation and the presence of anti-IgLON5 antibodies in serum and/or cerebrospinal fluid (CSF).

The precise functions of the antigen are unknown. The IgLON protein family belongs to the Immunoglobulin (Ig) protein superfamily and has three Ig-like domains, attaching to the membrane through a GPI-anchor. They are expressed in synapses and engage with each other forming homo- or heterodimers [11]. IgLON protein dimerization involves the first Ig-domain and is likely involved in neuronal outgrowth and synaptogenesis [11,12]. Autoantibodies directed toward IgLON5 bind specifically to an epitope in the second Ig-domain [13]. This binding is thought to be pathogenic and dependent on the IgG subclass. Anti-IgLON5 IgG1 has been shown to cause the irreversible internalization of IgLON5 clusters, whereas IgG4 may affect protein–protein interaction [13]. As such, the predominant subclass may affect the clinical presentation. The pathological properties of anti-IgLON5 antibodies were recently described in cultured hippocampal rat neurons with increased neurodegenerative features such as neuronal blebbing and fragmentation. However, in these cells, no hyperphosphorylation of tau or cell death was observed [14].

To further add to the link between neuroinflammation and neurodegeneration, we first studied the effects of short-term exposure (five days) of anti-IgLON5 IgG on neurons derived from human neural stem cells (hNSC). In this model, we found signs of neurodegeneration, including an increased number of p-tau (T205)-positive neurons, but no cell death. Consequently, we investigated the effects of long-term exposure (7–35 days) on neurons derived from human-induced pluripotent stem cells (hiPSCs) predifferentiated to the NSC stage. Here, we confirmed a similar accumulation of p-tau (T205) but also disruption of synapses and decreased neuronal spike activity. Finally, we detected increased cell death upon prolonged antibody exposure. Besides providing the first evidence of IgLON5 antibody effects in human neurons, these data also highlight the potential use of hiPSC models in the investigation of antibody-mediated diseases.

## 2. Materials and Methods

### 2.1. Isolation of Anti-IgLON5 IgG Fractions

The use of antibody samples was approved by the Ethical Committee at the Region of Southern Denmark (Approval No.: S-20170134). Serum samples containing both anti-IgLON5 IgG1 and IgG4 were obtained from a patient with a positive cell-based assay (CBA) and clinically verified anti-IgLON5 disease. The clinical features of the patient have been reported elsewhere [4]. Antibody binding was confirmed using indirect immunofluorescence on the primate cerebellum. Sera were analyzed in a 1:10 dilution and titrated to endpoint (1:1000). The presence of other encephalitis-causing autoantibodies was excluded by monospecific recombinant fixed commercial CBAs (NMDAR, LGI1, CASPR2, AMPAR1, AMPAR2, GABAbR, DPPX). IFA on monkey cerebellar sections (Euroimmun AG, Lübeck, Germany) showed blotchy fluorescence of the granular layer compatible with anti-IgLON5. Euroline Paraneoplastic Neurological Antigens 12 Profile line immunoassay (LIA) (Euroimmun AG, Luebeck, Germany) was negative (testing for amphiphysin, CV2, Ma/Ta, Ri, Yo, Hu, recoverin, Sox1, titin, Zic4, GAD, and Tr autoantibodies). Control human IgG was provided from sera of healthy donors and tested using commercial CBAs to exclude the presence of encephalitis-causing autoantibodies.

The IgG fraction of the patient and control serum were purified using a Protein A gel (MabSelect^TM^, Amersham Biosciences, Little Chalfont, UK) and then concentrated and transferred into sterile Phosphate-Buffered Saline (PBS) by centrifugation at room temperature (RT–3000× *g*), using protein concentrator tubes (VIVASPIN 20, Sartorius, Göttingen, Germany). Both purified IgG fractions from the patient and controls were retested on CBAs and the anti-IgLON5 IgG concentration was determined (titer 1:100). The antibody eluate was stored at −80 °C until usage.

### 2.2. Human Stem Cell-Derived Neurons (hNSCs and hiPSCs)

For short-term exposure experiments, hNS1, an immortalized human neural stem cell (hNSC) line (kindly provided by Dr. Alberto Martínez-Serrano, Autonomous University of Barcelona, Spain) was propagated and proliferated in a Poly-L-Lysine (10 µg/mL, Sigma-Aldrich, St. Louis, MO, USA)-coated T-25 flask (Nunc Easyflask, Nunc, Roskilde, Denmark) [15]. Cell propagation was performed using HNSC.100 media (composed of DMEM/F12, glucose, Hepes, AlbuMAX-I, N2 supplement, and nonessential amino acids (Gibco, Thermo Fisher, Waltham, MA, USA)), with the addition of pen/strep and the growth factors EGF and bFGF (20 ng/mL, R&D Systems, Minneapolis, MN, USA). The culture medium was changed every third day. Once an adequate confluence of 80–90% was reached, the cells were trypsinized using trypsin/EDTA (Gibco, Thermo Fisher, Waltham, MA, USA), and a single cell suspension was plated in 24-well plates (24 Well Cell Culture Cluster, Costar, Thermo Fisher, Waltham, MA, USA) with Poly-L-Lysine-coated 15 mm coverslips (Hounisen, Skanderborg Denmark) with a seeding density of 50,000 cells/cm^2^. Plates of monolayer cultures were incubated at 37 °C in a 5% CO_2_, 95% humidified air environment and differentiated for 14 days in HNSC.100 medium without growth factors. A 50% medium change was performed every third day. From Day 9 and five days forward, cultures were exposed to either IgG from a healthy control (1:50) or anti-IgLON5 IgG (1:50, Figure 1A).

For long-term exposure experiments, a predifferentiated human-induced pluripotent stem cell line (hiPSC, XCell Science, Novato, CA, USA) was propagated in Geltrex (Gibco, Thermo Fisher, Waltham, MA, USA)-coated 6-well plates [16]. Cell propagation was performed using NSC medium composed of neurobasal, B27, nonessential amino acids, and Glutamax (Gibco, Thermo Fisher, Waltham, MA, USA) with the addition of pen/strep and the growth factor bFGF 10 ng/mL. The medium was changed every third day, and cells were enzymatically passaged using Accutase (Gibco, Thermo Fisher, Waltham, MA, USA). Cells were differentiated in NSC medium without bFGF and passaged at Days 5 and 10. At differentiation on Day 10, cells were plated in both 24-well plates with Geltrex coated 15 mm coverslips or directly into 24-well plates for the multielectrode array (MEA) multiwell system. From Day 13, cultures were exposed to IgG from healthy controls (1:50) or anti-IgLON5 IgG (1:50, Figure 1B). Cells were exposed between 7 and 35 days in total.

### 2.3. IgLON5 Live Staining

Selected cultures were washed once in DMEM/F12 for hNSC cultures and neurobasal medium for hiPSC cultures followed by incubation with primary antibody eluate 1:50 (patient anti-IgLON5 IgG) for 30 min. After a second wash, cells were incubated with secondary antibody (Alexa 488, goat anti-human IgG, 1:500, Thermo Fisher, Waltham, MA, USA) for 30 min. A final washing step was performed, and cultures were fixed in 4% paraformaldehyde for 10 min, washed in D-PBS, and mounted with ProLong Diamond Antifade (Life Technologies, Carlsbad, CA, USA). Staining was performed after 5 days of antibody exposure for hNSC cultures and 7, 21, and 35 days of antibody exposure for hiPSC cultures.

### 2.4. Immunocytochemistry

After fixation, cultures were permeabilized with 0.1% saponin wash buffer and blocked with a 5% goat serum solution and incubated with antibodies against selected proteins overnight at 4 °C: Anti-β-Tubulin III (β-Tub III) (Sigma, Thermo Fisher, Waltham, MA, USA, T8660, mouse, 1:2000); anti-p-tau (Cell Signaling, Danvers, MA, USA, T205/E7D3E, Rabbit, 1:100); synaptophysin (Sigma, S5768, mouse, 1:200); PSD95 (Thermo Fisher, Waltham, MA, USA, 51-6900, rabbit, 1:1000). The following day, cells were washed and incubated with secondary antibodies dependent on the primary antibody (Alexa 555 (Thermo Fisher, Waltham, MA, USA, goat anti-mouse 1:500) and Alexa 488 (Thermo Fisher, Waltham, MA, USA, goat anti-rabbit 1:500)) for one to two hours at RT followed by washing steps. All cultures were counterstained with 10 μM dihydrochloride hydrate 4′,6-Diamidino-2-phenylindole (DAPI) solution for 10 min at RT, washed, and mounted with ProLong Diamond Antifade.

### 2.5. Image Acquisition and Analysis

Images were obtained using a fluorescent microscope (Olympus BX54, Tokyo, Japan), with a 60× objective for the live-cell and synapse stainings, and a 10× objective for other immunocytochemical stainings.

Image analysis was performed using ImageJ imaging software (https://imagej.nih.htmlgov/ij/index, v2.0.0-rc-69/1.52n). For the IgLON5 live-cell staining, unspecific background staining was removed before the images were made binary. An automatic particle analysis gave the total number of IgLON5 clusters per image. For the total nuclear count, a Gaussian blur filter was applied before images were made binary and a watershed application segregated the individual corpuscular elements. Particle analysis gave the final nuclear count. During cell counting, a cell counter plugin tool was used to assist manual counting of pyknotic and fragmented nuclei. For the analysis of neurodegeneration, neurons that contained multiple or localized swelling (blebbing) separate from a distally located growth cone, or fragmentation of the neuronal process, was counted. The percentage of neurons containing degenerative changes was calculated. In neurite outgrowth analysis, β-Tub III pictures were uploaded in the NeuronJ plugin application (https://imagescience.org/meijering/software/neuronj/, v1.4.3) as 8-bit images. Color balance was optimized and standardized for all images. Tracings were added for assessment of the number and length of neuronal neurites, expressed as pixel length.

For p-tau analysis of hNSC cultures and hiPSC cultures after 7 days of antibody exposure, the number of neurons containing accumulations of p-tau protein in processes were counted with the cell counter plugin tool. Due to the complexity of the hiPSC cultures after 21 days of antibody exposure, it was not possible to do manual cell counting. Instead, the intensity of p-tau staining was measured and normalized to the intensity of the β-Tub III in the same image. The p-tau expression was then normalized to the respective control group for the hiPSC for comparison of the different time points.

Quantifications of PSD95 and synaptophysin stainings were performed with the “Find Maxima” command in ImageJ.

### 2.6. Neuronal Spike Rate

Neuronal spike rates were measured using an MEA system (Multi Channel Systems, Reutlingen, Germany). Thresholds for spike detection were set to five times the standard deviation of the signal from each electrode calculated during the last five minutes before data acquisition. Cultures were measured for 20 min at each time point and data optimized by low- and high-frequency filters using the Multiwell Analyzer software v1.8.7.

### 2.7. Lactate Dehydrogenase (LDH) Assay

A commercially available absorbance-based LDH kit (Promega, Madison, WI, USA) was used according to the manufacturer’s description to measure the release of LDH to the culture medium as an indicator of cell death. Briefly, conditioned media from each sample was added to wells in a 96-well plate in combination with CytoTox 96 reagent, followed by 30 min of incubation in RT and protected from light. The reaction was stopped by the addition of stop solution to each well and absorbance was read at 490 nm to give an indirect measure of the amount of released LDH.

### 2.8. Statistical Analysis

Data were analyzed using a two-tailed unpaired *t*-test or two-way ANOVA, followed by Sidak’s multiple comparisons tests when appropriate. Values are presented as means ± SEM. A value of *p* < 0.05 was considered significant. Experiments were performed in independent duplicates or triplicates, and all *n*-values reported are the total number of wells analyzed. Five random images were collected from each well, and the average value was used for statistical analysis between groups. All statistical analyses were performed using GraphPad Prism v7.0.0 for Mac (GraphPad Software, San Diego, CA, USA, www.graphpad.com).

## 3. Results

### 3.1. Short-Term Autoantibody Exposure of hNSC-Derived Neural Cultures

**Figure 2:**
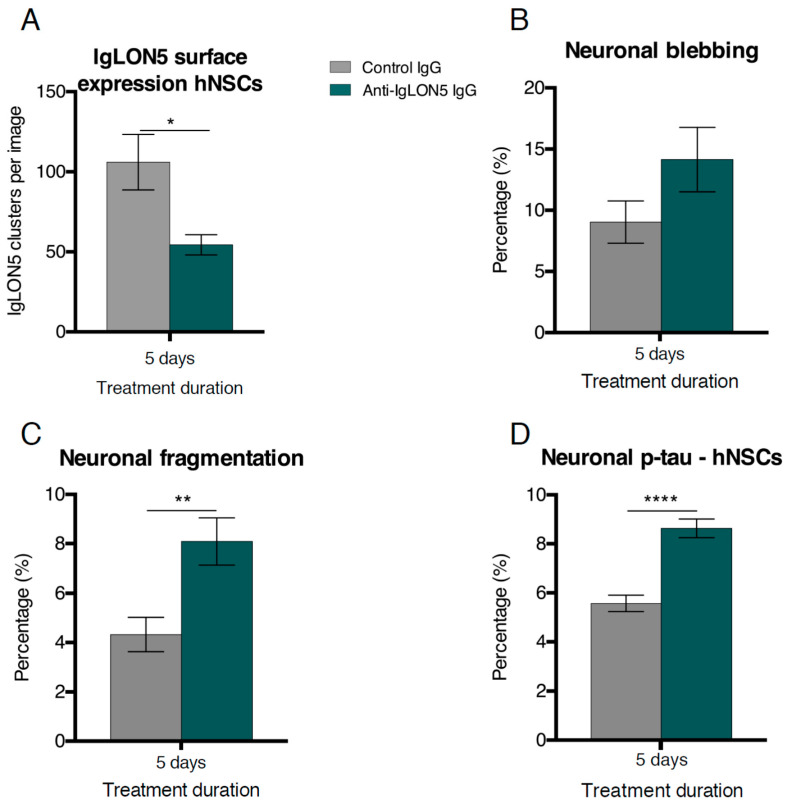
Anti-IgLON5 antibodies cause neurodegenerative changes in human neural stem cell derived (hNSC) neural cultures. Anti-IgLON5 antibodies significantly reduced the number of cell surface IgLON5 clusters (**A**) (*n* = 6). When evaluating degenerative changes in hNSC-derived cells after 5 days of antibody exposure, there was a tendency toward increased neuronal blebbing (**B**) (*n* = 18–19) when comparing anti-IgLON5 IgG to control IgG. There was also a significant difference in fragmented processes (**C**) (*n* = 18–19) between anti-IgLON5 IgG and Control IgG-treated groups. Cultures treated with anti-IgLON5 IgG showed an increase in the percentage of neurons with phosphorylated-tau (p-tau) (T205) when compared to cultures exposed to control IgG (**D**) (*n* = 18–19). Statistical method: two-tailed unpaired *t*-tests (* *p* < 0.05; ** *p* < 0.01; **** *p* < 0.0001).

As expected, anti-IgLON5 exposed cultures had a significant reduction of cell surface IgLON5 clusters compared to cultures exposed to control IgG (Control IgG: 106.0 ± 14 clusters per image, anti-IgLON5 IgG: 54.4 ± 7 clusters per image, *p* = 0.0187) (Figure 2A).

In addition, significantly more fragmentation of neuronal processes (IgLON5 IgG: 8.15 ± 0.71%; Control IgG: 4.3 ± 0.46%, *p* = 0.0030) and a tendency toward more blebbing processes (IgLON5 IgG 14.27 ± 1.40%; Control IgG 9.03 ± 0.92%, *p* = 0.1125) was found in anti-IgLON5 exposed cultures (Figure 2B,C). There were no differences in neurite number, neurite outgrowth length, or number of branch points (Supplementary Figure S1). In line with the neurodegenerative findings of fragmentation and blebbing, p-tau (T205) was higher in anti-IgLON5-treated cultures compared to controls (8.63 ± 0.30% and 5.57 ± 0.24%, respectively, *p* < 0.0001) (Figure 2D). As a measure of cell death, we quantified nuclei with either healthy or fragmented/pyknotic morphology. No differences in cell death were observed for hNSC cultures within the five days of exposure (Supplementary Figure S1).

In summary, short-term exposure to anti-IgLON5 IgG in hNSCs resulted in increased neurodegenerative features but no cell death. This was possibly due to the short exposure duration. To investigate the long-term effects of anti-IgLON5 on human neurons, we used hiPSC-derived neural cultures as they are not immortalized and considered a more accurate human cell model.

### 3.2. Long-Term Autoantibody Exposure of hiPSC-Derived Neural Cultures

**Figure 3:**
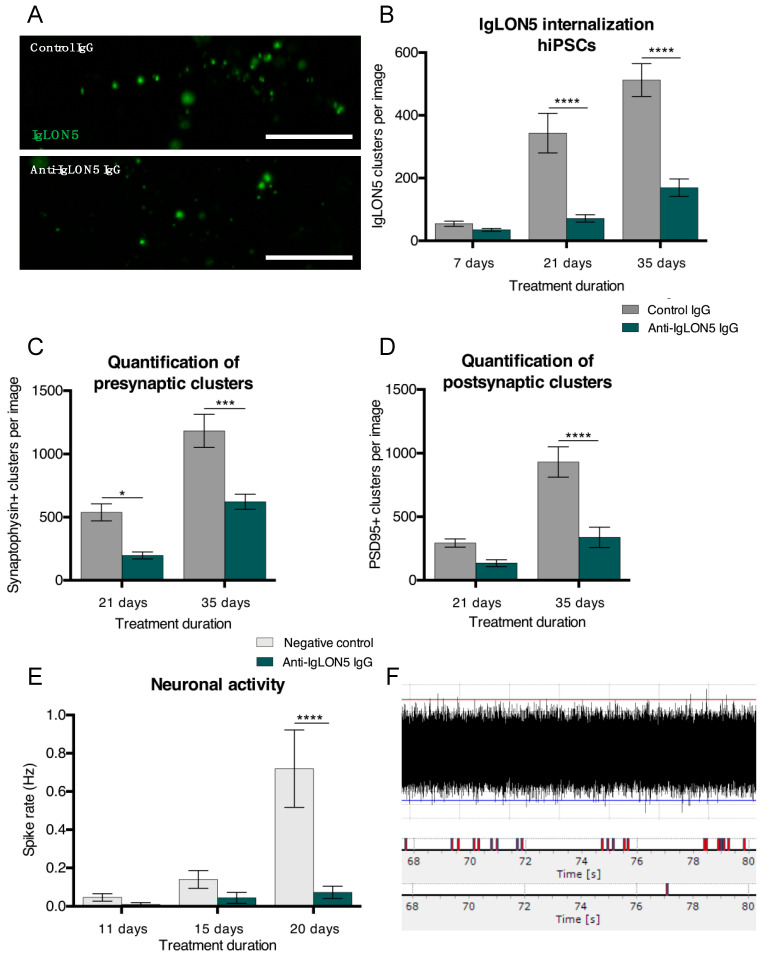
Anti-IgLON5 antibodies reduce cell surface IgLON5 clusters, synaptic proteins, and neuronal activity. Indirect immunofluorescent staining of human induced pluripotent stem cell (hiPSC) cultures treated with control IgG or anti-IgLON5 IgG (**A**). Quantification of IgLON5-positive clusters in hiPSC cultures revealed a significant reduction after 21 and 35 days of antibody exposure (**B**) (*n* = 9). Immunostaining for the synaptic proteins synaptophysin and PSD95 revealed a decrease in synaptophysin clusters after 21 days of exposure and in both synaptophysin and PSD95 clusters after 35 days of exposure (**C**,**D**) (*n* = 6). Multi electrode array (MEA) analysis of hiPSC cultures showed the expected increase in neuronal activity as they matured, however, the spike rate of neurons treated with anti-IgLON5 IgG was significantly lower than that of untreated neurons (**E**) (*n* = 5). Example of a MEA data sample is shown in (**F**) (top). When the signal crosses the threshold (horizontal lines) a spike is registered. Registered spikes from a single electrode in a well with an untreated culture (F, middle) and an anti-IgLON5 IgG-treated culture (F, bottom) during a 15 s period. Statistical analysis: two-tailed unpaired *t*-test or Two-way ANOVA followed by Sidak’s multiple comparisons test (*: *p* < 0.05, ***: *p* < 0.001, ****: *p* < 0.0001). Scale bar length: 5 μm.

Similar to hNSC-derived cultures, hiPSC-derived cultures exposed to anti-IgLON5 IgG had a significant reduction of cell surface IgLON5 clusters after 21 and 35 days of exposure compared to cultures exposed to control IgG (21 days: 343 ± 63 clusters vs. 71 ± 12 clusters, *p* < 0.0001; 35 days: 512 ± 52 clusters vs. 169 ± 28 clusters, *p* < 0.0001 (Figure 3A,B)). Further investigation of synaptic proteins revealed a reduction of presynaptic (synaptophysin) clusters after 21 days (465 ± 64 clusters vs. 208 ± 28 clusters, *p* = 0.0143), and both pre- and postsynaptic (PSD95) clusters after 35 days of exposure (1032 ± 116 presynaptic clusters vs. 917 ± 140 presynaptic clusters, *p* = 0.0002 and 603 ± 66 postsynaptic clusters vs. 274 ± 50 postsynaptic clusters, *p* < 0.0001 (Figure 3C,D)). To explore the possible consequences of these neurodegenerative features, we investigated the electrophysiological activity of our cultures. After 20 days, the spike rate of anti-IgLON5-exposed cultures was significantly reduced compared to controls (0.720 ± 0.203 Hz vs. 0.073 ± 0.032 Hz, *p* < 0.0001 (Figure 3E,F)).

**Figure 4:**
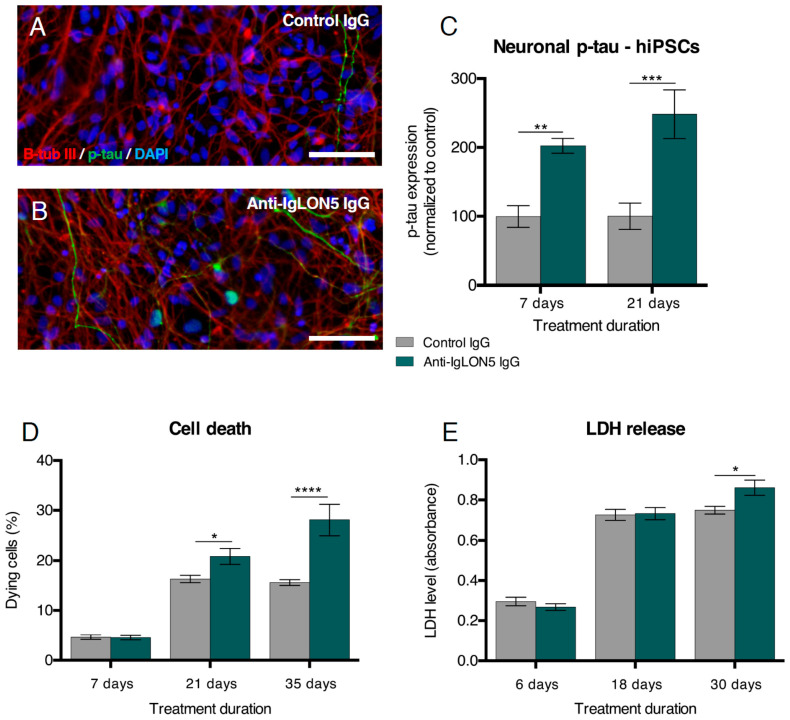
Anti-IgLON5 antibodies increase cell death in induced pluripotent stem cell (hiPSC)-derived neural cultures. Cultures treated with anti-IgLON5 IgG showed an increase in phosphorylated-tau (p-tau) (T205) expression after 7 and 21 days of exposure (**A**–**C**) (*n* = 7–8). Quantification of nuclei with unhealthy morphology revealed an increased percentage of dying cells after 21 and 35 days of exposure (**D**) (*n* = 7–18). A lactate dehydrogenase (LDH)-assay (a marker of necrosis) showed increased LDH release to the culture medium of anti-IgLON5 IgG exposed cultures after 30 days (**E**) (*n* = 8). Statistical analysis: two-way ANOVA followed by Sidak’s multiple comparisons test. (* *p* < 0.05, ** *p* < 0.01, *** *p* < 0.001, **** *p* < 0.0001). Scale bars: 50 μm. β-tub III: β-tubulin III.

When investigating the neurodegenerative features, we found that anti-IgLON5 IgG exposure increased p-tau (T205) expression in hiPSC-derived neurons after 7 (*p* = 0.0078) and 21 days (*p* = 0.0001 (Figure 4A–C)). Due to the increasing complexity of the cultures, it was not possible to accurately identify individual p-tau positive neurons after 35 days of exposure.

Similar to hNSC-derived cultures exposed to anti-IgLON5 IgG, we found no difference in cell death for hiPSC-derived cultures after 7 days of exposure. After 21 and 35 days, however, significant cell death was found (21 days: 16.3 ± 0.7% vs. 20.9 ± 1.6% abnormal nuclei, *p* = 0.0287. 35 days: 15.6 ± 0.6% vs. 31.1 ± 3.1% abnormal nuclei, *p* < 0.0001 (Figure 4D)). In accordance with this, the LDH release into the medium from anti-IgLON5 treated cultures was increased at Day 30, (*p* = 0.0140 (Figure 4E)).

## 4. Discussion

### 4.1. Effects of Anti-IgLON5 IgG Short-Term Exposure on hNSC-Derived Cultures

In this study, we describe the effects induced by anti-IgLON5 antibodies, providing the first demonstration of these effects on living human neurons.

In hNSC cultures, anti-IgLON5 IgG reduced the number of cell surface IgLON5 clusters. This is in accordance with previous reports on the pathological effects of patient anti-IgLON5 antibodies [13,14].

Axonal blebbing has been used to describe unhealthy neuronal morphology in primary hippocampal and cortical rat cultures and as a marker for chronic degenerative changes caused by neuroinflammation in in vivo murine models [17,18]. Neuronal cells with an unhealthy morphology were recognized in all anti-IgLON5 exposed hNSC-derived cultures, as we found cultures exposed to anti-IgLON5 IgG had a higher percentage of neurons with degenerative changes such as axonal blebbing and fragmentation. This is in line with recent observations in cultured rat hippocampal neurons and further support that exposure to anti-IgLON5 antibodies may cause secondary neurodegeneration [14]. Moreover, our exposed cells were also found to have smaller/pyknotic nuclei and were seen through a spectrum, where they eventually released their neuronal processes. It is tempting to speculate that these cells were destined to die and, if given more time, would add to the cell death in our anti-IgLON5 exposed cultures. Nevertheless, during this short-term exposure experiment, we found no increase in cell death in hNSC cultures treated with anti-IgLON5 IgG, which is likely due to a short experiment duration. Despite an unhealthy morphology, we did not find any differences in neurite number, length, and branch points in hNSC cultures treated with anti-IgLON5 IgG compared to controls. Whether this is due to a very short five-day antibody exposure period or because IgLON5 does not regulate neurite outgrowth remains unclear.

The content of neuronal p-tau (T205) was higher in cultures exposed to anti-IgLON5 IgG. Accumulation of p-tau is a characteristic feature of neurodegeneration and patients with anti-IgLON5 disease are found to have p-tau accumulation in postmortem examinations [2,19]. Our finding supports the hypothesis that neuroinflammation precedes neurodegeneration and that anti-IgLON5 antibodies may contribute to the neurodegenerative changes found in patients with anti-IgLON5 disease. Further studies on human neurons with antibodies from more patients and investigation of other p-tau forms are, however, needed to clarify this.

### 4.2. Effects of Anti-IgLON5 IgG Long-Term Exposure on hiPSC-Derived Cultures

Similar to the hNSC cultures, we found that anti-IgLON5 IgG reduced the amount of cell surface IgLON5 clusters in hiPSC cultures. Because IgLON proteins are thought to be involved in synaptogenesis, we quantified the amount of post- and presynaptic proteins PSD95 and synaptophysin. Both were found to be decreased in cultures exposed to anti-IgLON5 IgG. Whether this is caused by synapse disruption by IgLON5 antibodies, reduced neuronal maturation, or part of early neurodegeneration is unknown. To investigate the potential effects of this, we examined the electrophysiological properties of anti-IgLON5 exposed cultures and found reduced electrophysiological activity of these neurons. Although this is a novel observation of anti-IgLON5 antibodies, changes in electrophysiological properties are a very commonly described effect of other autoimmune encephalitides, likely due to the synaptic localization of many described antigens [20,21,22].

Increased p-tau (T205) accumulation was seen in hiPSC cultures early after IgLON5 antibody exposure (7 days) and further increased after 21 days, confirming our results from the hNSC cultures. Similar to the hNSC cultures, we found no difference in cell death in hiPSC cultures after seven days of exposure. However, at 21 and 35 days, increased cell death was observed accompanied by an increased number of fragmented and pyknotic nuclei and increased LDH release to the medium. To our knowledge, this is the first demonstration of antibody-mediated degenerative changes in cultures of human neurons and supports that the autoantibodies in anti-IgLON5 disease are causative for the disease and not a secondary event.

Our study is limited by the use of anti-IgLON5 from a single patient. Anti-IgLON5 disease is, however, rare, with an estimated incidence of 1/150,000 and with only two cases documented in Denmark [4]. Although our data are indicative of IgLON5-antibody-induced neurodegeneration, further studies on human neurons with samples from more patients and the addition of different neuronal surface antibodies would be of value to validate the specificity. In addition, investigation of other p-tau forms would be of great value in studies of cellular mechanisms forthcoming.

## 5. Conclusions

The data in this study indicate several novel findings: (1) Anti-IgLON5 antibodies may cause a decrease in neuronal communication; (2) cultures of human neurons exposed to patient anti-IgLON5 IgG exhibited increased neurodegenerative changes with axonal fragmentation and increased p-tau (T205) expression; and (3) exposure to anti-IgLON5 antibodies leads to increased cell death. These findings support the hypothesis that anti-IgLON5 antibodies lead to neurodegeneration. They also stand as a proof-of-concept of the potential of human stem cells and especially hiPSCs for research in antibody-mediated neurological diseases. As stated above, further studies are, however, needed to validate these findings.

## Figures and Tables

**Figure 1:**
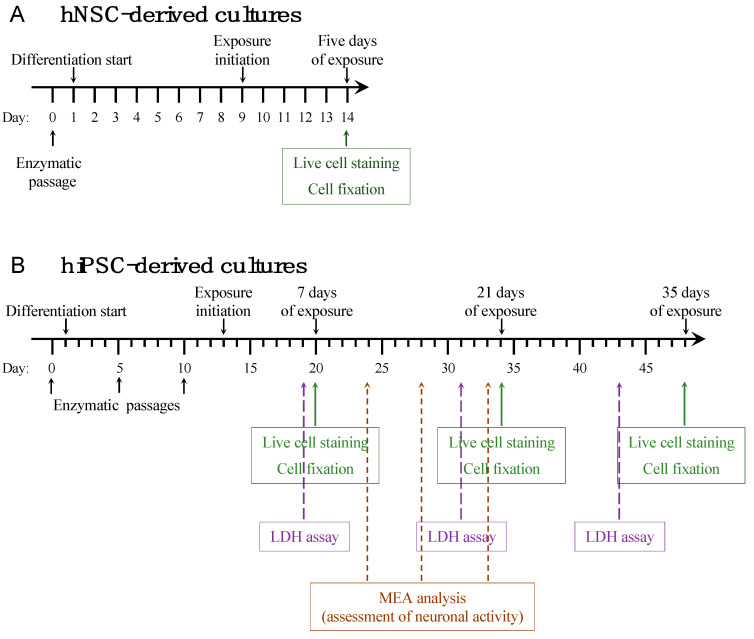
Graphical illustration of the experimental setup. Short-term autoantibody exposure was performed on human Neuronal Stem cells (hNSC) and the experimental setup is shown in panel (**A**). Long-term autoantibody exposure was performed on human-induced Pluripotent Stem Cell (hiPSC)-derived neurons and the experimental setup is shown in panel (**B**). Black arrows and text specify differentiation procedures. Colored arrows and text boxes specify the time points at which different analyses were performed. LDH: lactate dehydrogenase, MEA: multi electrode array.

## Data Availability

Data supporting the findings of this study are available within the figures and the results section. Further data and extended description of methods are available from the corresponding author upon reasonable request.

## Supplementary Materials

The following are available online at https://www.mdpi.com/article/10.3390/cells10040837/s1, Figure S1: Cultures treated with anti-IgLON5 IgG or control IgG had similar number of neurites (A) (*n* = 6), average neurite length (B) (*n* = 6), number of branch points (C) (*n* = 6) and percentage of dead or dying cells (D) (*n* = 6) after 5 days of antibody exposure. There was no significant difference between expression of beta-tubulin III in hNSC (E) (*n* = 6) or hIPSC (F) (*n* = 6) cultures.
