# Supplementary material for: Neurodegeneration Induced by Anti-IgLON5 Antibodies Studied in Induced Pluripotent Stem Cell-Derived Human Neurons
Source: Cells. 2021 Apr 8;10(4):837. doi: 10.3390/cells10040837 (PMC8068068; doi:10.3390/cells10040837)
Supplement: Supplementary file 1 [file cells-10-00837-s001.pdf]

cells MDPI

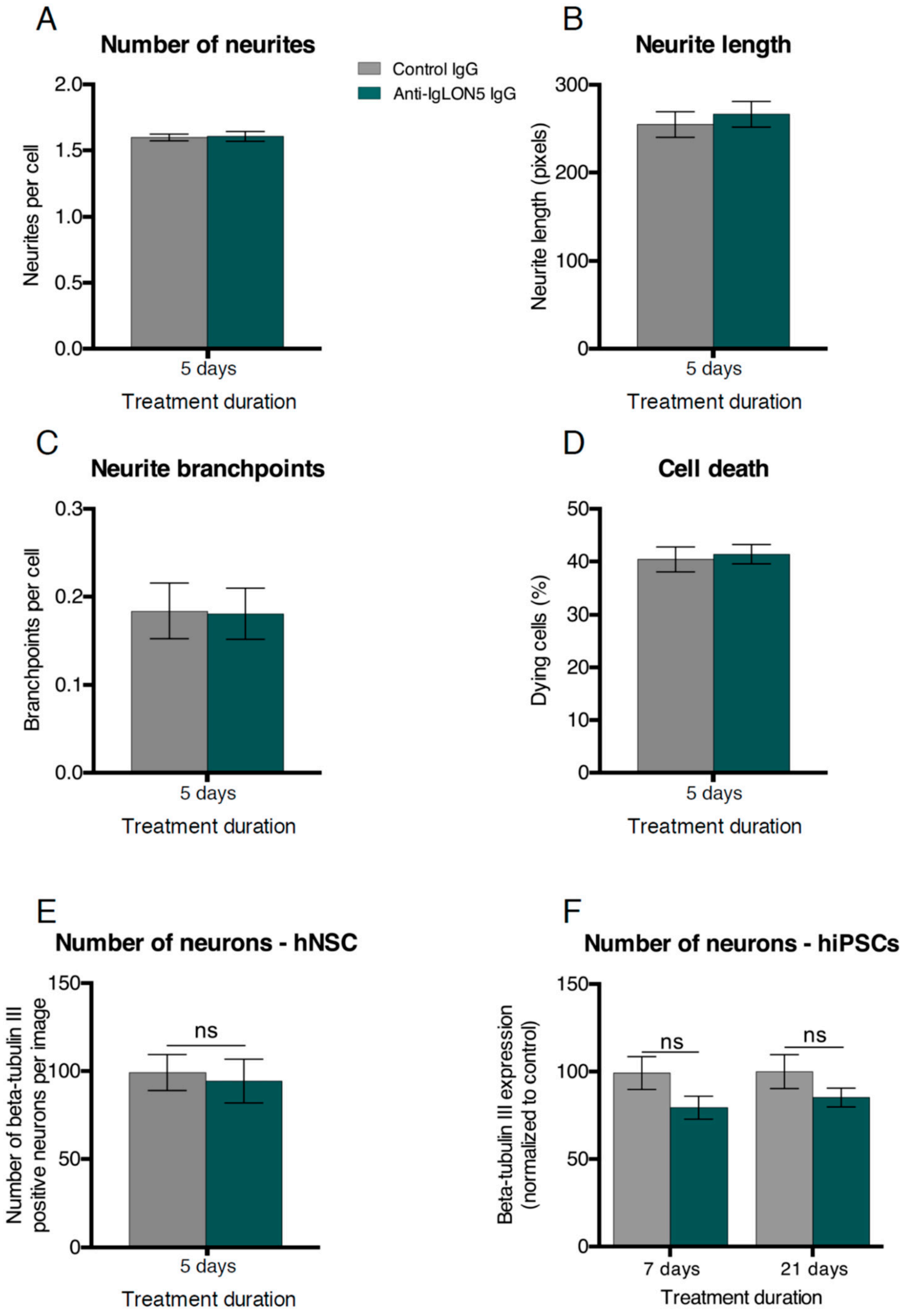

Supplementary Figure S1. Cultures treated with anti-IgLON5 IgG or control IgG had a similar number of neurites (**A**) (n = 6), average neurite length (**B**) (n = 6), number of branchpoints (**C**) (n = 6), and percentage of dead or dying cells (**D**) (n = 6) after 5 days of antibody exposure. There was no significant difference between the number of beta-tubulin III-positive neurons in human neural stem cell-derived (hNSC) neurons (**E**) (n = 6) or human induced pleuripoten stem cell-derived (hIPSC) neurons (**F**) (n = 6) cultures. n.s = not significant
